# A Promising Wearable Solution for the Practical and Accurate Monitoring of Low Back Loading in Manual Material Handling

**DOI:** 10.3390/s21020340

**Published:** 2021-01-06

**Authors:** Emily S. Matijevich, Peter Volgyesi, Karl E. Zelik

**Affiliations:** 1 Department of Mechanical Engineering, Vanderbilt University, Nashville, TN 37212, USA; karl.zelik@vanderbilt.edu; 2 Institute for Software Integrated Systems, Vanderbilt University, Nashville, TN 37212, USA; peter.volgyesi@vanderbilt.edu; 3 Department of Biomedical Engineering, Vanderbilt University, Nashville, TN 37232, USA; 4 Department of Physical Medicine & Rehabilitation, Vanderbilt University, Nashville, TN 37232, USA

**Keywords:** overexertion injury, ergonomics, machine learning, lumbar moment, risk assessment, wearables, fatigue failure, lifting biomechanics

## Abstract

(1) Background: Low back disorders are a leading cause of missed work and physical disability in manual material handling due to repetitive lumbar loading and overexertion. Ergonomic assessments are often performed to understand and mitigate the risk of musculoskeletal overexertion injuries. Wearable sensor solutions for monitoring low back loading have the potential to improve the quality, quantity, and efficiency of ergonomic assessments and to expand opportunities for the personalized, continuous monitoring of overexertion injury risk. However, existing wearable solutions using a single inertial measurement unit (IMU) are limited in how accurately they can estimate back loading when objects of varying mass are handled, and alternative solutions in the scientific literature require so many distributed sensors that they are impractical for widespread workplace implementation. We therefore explored new ways to accurately monitor low back loading using a small number of wearable sensors. (2) Methods: We synchronously collected data from laboratory instrumentation and wearable sensors to analyze 10 individuals each performing about 400 different material handling tasks. We explored dozens of candidate solutions that used IMUs on various body locations and/or pressure insoles. (3) Results: We found that the two key sensors for accurately monitoring low back loading are a trunk IMU and pressure insoles. Using signals from these two sensors together with a Gradient Boosted Decision Tree algorithm has the potential to provide a practical (relatively few sensors), accurate (up to r^2^ = 0.89), and automated way (using wearables) to monitor time series lumbar moments across a broad range of material handling tasks. The trunk IMU could be replaced by thigh IMUs, or a pelvis IMU, without sacrificing much accuracy, but there was no practical substitute for the pressure insoles. The key to realizing accurate lumbar load estimates with this approach in the real world will be optimizing force estimates from pressure insoles. (4) Conclusions: Here, we present a promising wearable solution for the practical, automated, and accurate monitoring of low back loading during manual material handling.

## 1. Introduction

Low back disorders are a leading occupational health problem, ranging from lumbar (low back) pain to muscle strains to herniated spinal discs. Physical pain, missed work, decreased productivity, healthcare costs, short- and long-term disability, and psychological distress due to these low back disorders are substantial and persistent burdens on our society. Back disorders account for about 40% of all work-related musculoskeletal disorders [1], and about one in four workers reports dealing with low back pain [2,3]. Individuals working in manual material handling jobs (and other jobs with similar physical demands) are at particularly high risk for low back disorders due to repetitive lifting and bending, which can lead to musculoskeletal overexertion (overuse) injuries [1].

Overexertion injuries result from an accumulation of microdamage caused by repetitive loading to musculoskeletal tissues (e.g., muscles, tendons, ligaments, bones, discs). Overexertion injuries are consistent with a fatigue failure process: the weakening and eventual failure of a material due to repeated loading [4,5,6]. When modeling this fatigue failure process, both the number of loading repetitions and the magnitude of loading on the musculoskeletal tissues are important for approximating the cumulative damage to the tissues. There are multiple opportunities to use musculoskeletal loading and fatigue failure insights to understand and reduce the risk of overexertion injuries, such as through ergonomic assessments or continuous, personal monitoring of injury risk.

### 1.1. Ergonomic Assessments

Ergonomic risk assessment tools that evaluate low back loading and assess injury risk using fatigue failure principles have shown potential for predicting the incidence of low back disorders. For example, the Lifting Fatigue Failure Tool (LiFFT) estimates cumulative tissue damage to the low back using an estimate of lumbar moment. Cumulative damage across a series of lifting tasks estimated with LiFFT has been shown to explain 72–95% of the deviance in low back disorders from epidemiological databases [4]. Ergonomic risk assessments are traditionally performed via direct observation by a trained professional. For instance, to perform an ergonomic assessment using LiFFT (or other assessment tools like the NIOSH Lifting Equation [7]), an ergonomist or safety professional would monitor a single worker during their shift, or over a subset of representative job tasks, to manually record how much each lifted object weighed and how far away each lifted object was from the body, then input how many times each type of lift is performed during a shift. The time spent observing a worker depends on the variability of job tasks (e.g., short- vs. long-cycle jobs), but is often on the order of 1–8 h per job.

While these valuable ergonomic assessments and injury risk profiles can inform the use of ergonomic controls to minimize the risk to workers, the assessments can be time-consuming and costly. Assessments can become prohibitively expensive when there are a large variety of jobs at a given workplace or when job functions are remote, unobservable, highly variable, or infrequent. Moreover, this kind of time-intensive professional observation is impractical for personalized, continuous monitoring of injury risk over long durations or across an entire workforce. Video-based solutions that leverage advances in computer vision and machine learning have the potential to address some of these challenges by providing a semi-automated analysis of jobs. However, this approach is impractical for highly dynamic jobs (e.g., a construction worker moving all over a construction site), or jobs where visual obstructions occur (e.g., an aerial porter climbing in and out of arriving planes) and is not intended for personalized monitoring across an entire workforce. To efficiently evaluate ergonomic risk across a wide range of workers, high-risk jobs, and workplace environments, there remains a need for tools that enable the automated, unconstrained, and widespread monitoring of musculoskeletal loading and damage, particularly to the lower back.

### 1.2. Wearable Sensors at a Single Body Location for Ergonomic Assessment or Continuous Monitoring

Small, inexpensive, wearable sensors offer a promising solution for the unconstrained monitoring of job demands, including in confined spaces or during dynamic jobs. Wearable solutions could automate traditional job analysis or ergonomic assessments by replacing time-consuming observations and manual measurements with automated analytics from wearable sensor data, potentially improving the quality (e.g., consistency, accuracy) and quantity of data (e.g., the amount of assessment time per worker, the number of workers evaluated). Further, wearables can be practical for continuous monitoring, providing new opportunities to perform ergonomics assessments for remote and long-cycle duration jobs or for personalized, daily injury risk monitoring that could inform ergonomic controls. Continuous monitoring also has the long-term potential to usher in a new era of preventative occupational safety and health that transforms how musculoskeletal risk is managed and insured.

While wearable sensors offer an exciting tool for monitoring low back loading and overexertion risks, current commercial and research technologies have some key limitations. Current commercial products (e.g., StrongArm Fuse, Soter Analytics Clip&Go, Kinetic Reflex, and Modjoul Smartbelt) use a single inertial measurement unit (IMU) mounted on the waist, back, or chest and analyze motion data (e.g., trunk orientation or acceleration) and the frequency of lifting/bending. We refer to these types of devices as wearable sensors at a single body location (or single wearable solutions, for short). We use this terminology because they each use hardware placed on one body location, although this hardware unit may contain multiple different sensors that measure numerous signals (e.g., IMUs are generally composed of accelerometers, gyroscopes, and magnetometers).

These single wearable solutions are relatively practical to implement in the workplace and may be most amenable to job analyses that characterizes postures and task frequency, but less well-suited for ergonomic assessments that quantitatively assess injury risk based on musculoskeletal loading and fatigue failure principles. This is because low back loading is dependent on factors beyond the kinematics of a single body segment, including the mass of the object being lifted and how far away the object is from the body. So, while these single wearable solutions can use segmental motion data to identify when a worker performs a deep forward bend, they are generally unable to distinguish, for instance, if the worker lifted a 5 lb box vs. a 45 lb box. The heavier mass in this example is expected to result in 65× more tissue damage (based on LiFFT, and assuming boxes are located 25 inches anterior to the lumbar spine). There are some use cases where single wearable solutions are expected to estimate low back loading fairly well (e.g., if the objects lifted are of known mass and are in a fairly consistent location relative to the body). However, there are cases where single wearable solutions are likely to be insufficient because they do not account for varying object masses, object locations, or other external forces on the body. In these cases, single wearable solutions could potentially provide inaccurate or misleading information about loading and cumulative damage to the low back, or unreliable insight on low back injury risk for a specific job, task, or worker.

### 1.3. Distributed Wearable Sensors for Ergonomic Assessment or Continuous Monitoring

Using multiple wearable sensors at distributed locations on the body has the potential to provide better estimates of low back loading by capturing and integrating additional dynamics data (e.g., body segment motions or orientations, forces or moments, muscle activity). These distributed sensor solutions are conceptually similar to what is done in motion analysis labs when data from cameras, force plates, and/or other measurement modalities are combined with biomechanical models to compute the loading on the back. An example of a commercial distributed wearable sensor system is the Xsens system that uses up to 17 IMUs on different body segments to track motion. These data can then be passed through analytics software (e.g., Scalefit, AnyBody) to estimate musculoskeletal loading on the back. However, similar to single sensor solutions, distributed IMU systems cannot automatically distinguish the mass of the object being lifted. Often, this additional information must be entered manually, or additional sensor modalities must be added, which increases the complexity of data collection and analysis. Thus, distributed IMU systems may only partially automate ergonomic assessments, or they may provide inaccurate estimates of low back loading if analytics software simply assume a default object mass.

To fully automate back load monitoring, several research studies suggest that adding force-instrumented shoes or pressure insoles along with distributed IMUs over the upper and/or lower body is promising [8,9,10,11]. For instance, [8] showed that by combining 8–17 IMUs and force-sensing shoes, lumbar moments could be estimated within 10–20% of peak extension moments. [9] found corroborating results, showing that with 12 IMUs and pressure insoles the peak axial load on the L5/S1 joint could be estimated with errors <5%.

However, there are a couple of critical limitations of these approaches. First, many of these solutions were developed and evaluated on a limited range of manual material handling tasks. For instance, [8] only evaluated four lifting tasks, all with a 10 kg box. It therefore remains unclear if these combinations of wearable sensors and/or algorithms are accurate and generalizable to a broad range of complex manual material handling motions, as performed in real world environments. Second, these wearable solutions require a large number of sensors distributed across the body, which introduces practical challenges related to technology implementation, ease of use, acceptance, and adoption. For scientific research or infrequent ergonomic assessment, the burden of distributed instrumentation may be an acceptable trade-off for increased accuracy. However, using numerous sensors requires longer donning and doffing times and more complexity, which presents a pragmatic barrier for workplace adoption. To enable more efficient and widespread ergonomic assessments or continuous monitoring of injury risk, there remains a need for a solution that requires a smaller number of wearable sensors (to be practical) and provides validated estimates of low back loading for a wide range of work-relevant tasks (to ensure accuracy).

### 1.4. Key Requirements for Wearable Ergonomic Assessment: Practical and Accurate

Based on our review of commercial technologies and scientific literature and our conversations and observations with manual material handlers and safety professionals across a range of industries (e.g., logistics, manufacturing, retail, agriculture, construction, military), we identified what we believe to be a key technological gap and unmet industry need related to ergonomic assessment and continuous personal monitoring of low back overexertion injury risk. Specifically, we found that a portable wearable sensor tool with the following characteristics and capabilities does not currently exist, but if it did we believe it could be game-changing for low back injury risk assessment, monitoring, and prevention in various industries:

1. The tool is **practical** to don, doff, and wear in unconstrained environments for prolonged periods of time by virtue of using only a small number of sensors at different body locations. This is important for industrial acceptance, adoption, and implementation. Of note, there is no simple limit for the maximum number of sensors or body locations that is practical, but this consideration helped motivate the approach we took in this research (as detailed in Methods).
2. The tool provides **accurate**, validated, and automated estimates of low back loading for a broad range of manual material handling tasks. This is important to ensure the system will be reliable during use in the real world and can distinguish differences in back loading that result from lifting objects of different weights without the need for professional observation or manually inputting object weights or other data.

The overarching question we sought to address in this study was: if we can only use a small number of wearable sensors to monitor low back loading, then which sensors should we use, where should we place them, what type of algorithm should we employ to fuse the sensor data, and how accurately can we monitor low back loading during manual material handling tasks? To address this exploratory, multi-faceted, open-ended question, we collected synchronized data from laboratory instrumentation and wearable sensors across a broad range of lifting tasks and combined domain expertise in biomechanics with techniques from machine learning to develop musculoskeletal load estimation algorithms, similar to the approach we previously took to develop a wearable sensor system for monitoring bone loading and overexertion injury risks in the legs of runners [12]. The Methods section provides details on our exploratory approach and rationale.

## 2. Materials and Methods

### 2.1. Summary of Approach

Here, we briefly summarize our exploratory research approach, followed by detailed methodology below:

First, we identified a candidate set of wearable sensors (number, type, and location of sensors). We bounded our candidate sensors based on biomechanical insight, prior literature [8,9,10,11], and expected practicality for implementation in the real world. We selected IMUs placed on body segments (feet, shanks, thighs, pelvis, trunk) and pressure insoles placed inside the shoes (capable of estimating the interaction force and center of pressure between the foot and shoe) as our candidate sensors. These types of sensors are mature, and for years have been used in clinical and consumer devices that are worn daily; for instance, IMUs are ubiquitous in fitness trackers and phones, and pressure insoles are used for clinical screening (e.g., Orpyx) and to track running/sport performance (e.g., ARION, ReTiSense, NURVV). We elected not to use surface electromyography (EMG) due to practical challenges of implementing in the real world, such as their sensitivity to sweat, hair, and sensor placement, and reliability issues over days/weeks [13]. We also elected not to use any implantable or percutaneous sensors, or any emerging sensor technologies that have not yet been proven to be practical, reliable, affordable, and scalable in the real world. Focusing on mature, proven sensor technologies was with the hope and intention of arriving at a solution that would be feasible to translate into a product for real world use in the near future (e.g., next 2–5 years).

Second, we synchronously collected data from lab-based instrumentation and from real wearable sensors across 10 participants each performing about 400 different manual material handling tasks, which encompassed many different postures, movements, and object masses that a worker may encounter in the real world.

Third, we developed wearable sensor algorithms using various combinations of wearable sensor signals (algorithm inputs) and our lab-based gold-standard estimates of low back loading (algorithm target). We first used *idealized wearable sensor signals* [12], which consisted of lab-based data we converted into the types of signals reasonably obtained with wearables, to develop and evaluate algorithms. An example of an idealized wearable sensor signal is that we mapped the three-dimensional ground reaction force (GRF) vector from an in-ground force plate onto a one-dimensional force normal to the bottom of the foot to represent the type of signal that can be estimated from a pressure insole. This allowed us to explore algorithms for low back load estimation without worrying if the sensor or signal quality from a particular wearable sensor we used was a limiting factor. Next, we used *real wearable sensor signals* to separately develop and evaluate algorithms, benchmark the accuracy of current wearable sensor technologies, and assess how these may or may not limit low back load monitoring tools. We use the terminology *idealized wearable sensor signals* and *real wearable sensor signals* to distinguish these two complementary approaches. Throughout, we also use the terms *idealized wearable sensors* and *real wearable sensors* to refer to physical sensors or sensor combinations, with *idealized wearable sensors* referring to the sensors that would be needed to measure the particular signals used. See [12] (as well as the Discussion of this paper) for more rationale on the value of using idealized wearable sensor signals when exploring new solutions for musculoskeletal load monitoring.

Finally, by applying various machine learning techniques to various subsets of idealized and real wearable sensor signals, we: (1) quantified how the number of sensors used influenced the algorithm estimation accuracy, (2) identified the most important types and locations of sensors for low back load estimation, and (3) benchmarked how much using real vs. idealized wearable sensor signals influenced the estimation accuracy. Below, we describe the human participant experiment and data analysis, followed by algorithm exploration, development, and evaluation.

### 2.2. Experiment Overview

Ten healthy individuals participated in the study: 3 females and 7 males (age: 25 ± 3 years; height: 1.8 ± 0.1 m; mass: 79 ± 14 kg). All the participants gave written informed consent to the protocol, which was approved by the Institutional Review Board at Vanderbilt University (IRB # 141697).

**Figure 1:**
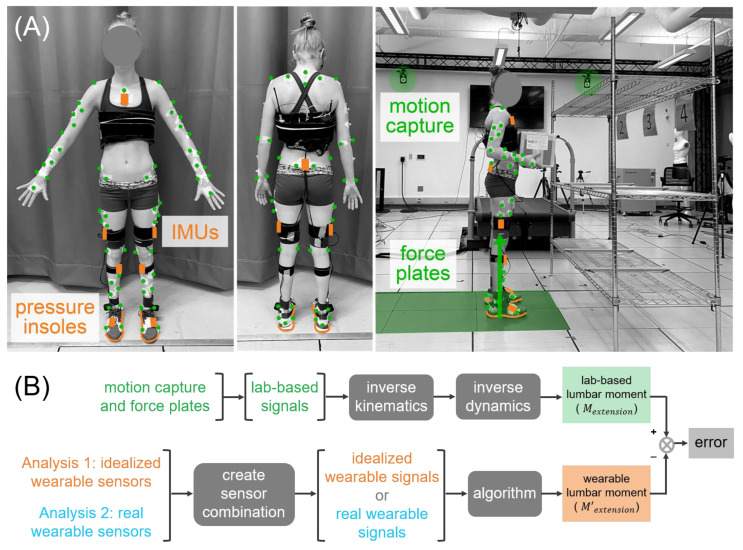
Experimentation and wearable algorithm development overview. (**A**) Lab-based (green) and wearable sensor (orange) signals were collected synchronously in a motion analysis lab while participants performed about 400 manual material handling tasks. (**B**) Lab-based analysis yielded a gold-standard estimate of lumbar extension moment (*M_extension_*). Wearable signal analysis and algorithm development yielded wearable sensor estimates of lumbar extension moment (*M’_extension_*). The wearable algorithm development was conducted twice, once using idealized wearable sensors (defined in Methods) as inputs (Analysis 1) and once using real wearable sensors as inputs (Analysis 2).

This study involved participants each performing about 400 manual material handling tasks in a motion analysis lab. Tasks covered a broad range of bending, turning, twisting, squatting, stooping, and reaching postures while lifting and moving boxes of 5–23 kg, which were representative of tasks commonly performed by manual material handlers (e.g., case pickers in a warehouse, retail workers stocking shelves, or logistics workers at a sort facility). For instance, tasks involved moving boxes from high to low shelves, low to high shelves, from a lateral to a forward position, diagonally between shelves, and much more to obtain a rich, diverse, realistic, and work-relevant data set (see Video S1 for example videos of tasks). The data collection space was outfitted with various shelves at 3 heights with labeled locations (see Figure 1A for an example setup). Box masses, shelf heights, and actions were informed by manual lifting and ergonomics guidelines [14]. For each task, participants were given instructions such as “move the box from position 3 to 4” (Figure 1A) and were told to use any safe strategy to complete the task. Each task was performed once and the participants were given rest breaks intermittently throughout the protocol.

#### 2.2.1. Lab-Based Measurement Modalities

We collected full-body kinematics and ground reaction forces (GRFs). Kinematics were collected at 200 Hz (Vicon), then low pass-filtered at 6 Hz (3rd order, zero-lag Butterworth). Four markers were placed on each thigh, shank, arm, and forearm; 5 markers were placed on each foot; 6 markers were placed on the pelvis; and 4 were placed on the trunk. Additional markers were placed on the lateral and medial femoral epicondyles, the lateral and medial malleoli, each acromion, the lateral and medial humeral epicondyles, and the distal radius and ulna. The GRFs under each foot were collected at 1000 Hz using in-ground force plates (AMTI). The GRFs were low pass-filtered at 10 Hz (3rd order, zero-lag Butterworth).

#### 2.2.2. Wearable Measurement Modalities

We synchronously collected IMU-based lower body and trunk kinematics (Xsens) and plantar pressures (Novel pedar-x, with 99 pressure sensors per insole). Kinematics were collected at 100 Hz using the standard Xsens “lower body + trunk” configuration and IMUs were oriented according to the Xsens participant preparation guidelines. Scaling, calibration, and data pre-processing were performed by the Xsens software, providing a built-in anatomical model. Plantar pressures were collected bilaterally at 100 Hz and the total (normal) force and center of pressure were exported using the Novel software. The synchronization of all measurement modalities was achieved through recorded analog triggers, and any delays between measurement modalities were accounted for through temporal alignment/calibration algorithms based on pilot testing.

### 2.3. Wearable Algorithm Development

A visual overview of the lab-based data analysis and algorithm evaluation workflow is provided in Figure 1B.

#### 2.3.1. Lab-Based Data Analysis (Algorithm Target)

We selected lumbar extension moment as our target musculoskeletal loading metric because it can be used to estimate cumulative tissue damage to the low back using a fatigue failure analysis [4,5,15]. We sought to estimate the time series lumbar extension moment (as opposed to just peak moments) because this enables us to identify bending/lifting frequency, to partition out individual movement cycles, and to better understand and distinguish cyclic lifts vs. prolonged bending. Time series data enables the assessment of loading and cumulative risk across all tasks, as well as the ability to perform task-specific load and risk assessment.

Lower-body segmental and joint kinematics were estimated based on optical motion capture data and rigid-body inverse kinematics. GRF and kinematics were combined via rigid-body inverse dynamics to estimate joint kinetics (C-Motion, Visual3D). Time series lab-based lumbar moment was estimated using bottom-up inverse dynamics in Visual3D. Moments are reported in units of body weight × body height (BW × BH).

#### 2.3.2. Wearable Sensor Signal Data Preparation (Algorithm Inputs)

We used time series wearable sensor signals as inputs to the algorithm. *Idealized wearable sensor* signals are summarized in Table 1. *Real wearable sensor* signals are summarized in Table 2. The algorithm development workflow was completed twice, once using idealized wearable sensor signals as the inputs and once using real wearable sensor signals as the inputs (Analysis 1 and Analysis 2, Figure 1B). The lab-based target, idealized wearable sensor signals, and real wearable sensor signals were all resampled to 100 Hz. Input signals were normalized to z-scores during the algorithm development [16].

#### 2.3.3. Algorithm Development

We explored supervised machine learning algorithms (e.g., generalized linear models, support vector machines, neural networks) for multiple variable regression to predict the lumbar extension moment (*M_extension_*) focusing on techniques that could provide instantaneous predictions, where wearable signals from a given time sample are used to estimate the target load metric for that same time sample. Ultimately, we achieved the most promising results with Gradient Boosted Decision Trees, a popular technique in machine learning and well-suited to handle missing values and redundant or non-predictive inputs [17,18]. The number of input signals (tens or hundreds) also fits this approach. Furthermore, by using a histogram-based decision tree building algorithm influenced by LightGBM [19], we dramatically decreased the algorithm training time (to a few seconds with a few million time samples) without a noticeable degradation in the prediction accuracy. Briefly, this algorithm estimates the target load metric by building an ensemble of decision trees in a stage-wise fashion, where in each stage the new tree tries to estimate (and thus, remove) the residual error after combining the predictions of the previous trees. Our current results are based on ensembles of approximately 100 trees. We used the scikit-learn library and Amazon SageMaker, a cloud-based machine learning platform for algorithm development, model training, and evaluation.

To develop the algorithm, we used k-fold validation by participant (*n* = 10), a commonly used technique to assess the generalizability of an algorithm [16]. In other words, we used data from nine participants to train the algorithm (i.e., select hyperparameters), and then evaluated the algorithm accuracy on data from the remaining participant. This process was repeated for all ten participants to yield wearable algorithm estimates of the lumbar extension moment (*M’_extension_*) for the entire dataset.

**Figure A1:**
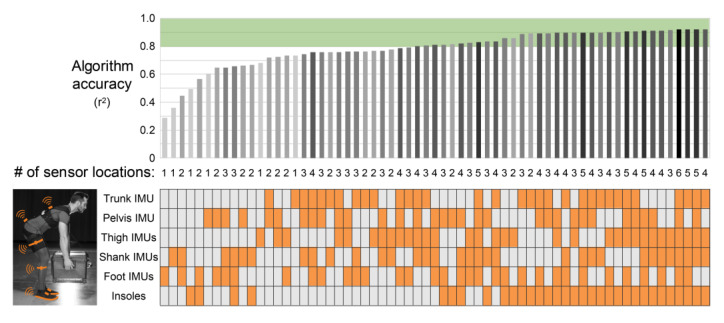
Number of sensor locations and specific sensor combination influences algorithm accuracy. (**Top**) Average accuracy using idealized wearable sensors summarized here using the average coefficient of determination (r^2^). Darker color bars correspond to an increasing number of sensor locations used in the algorithm. (**Bottom**) Summary of which sensor locations were used in each reduced sensor combination. Orange grid boxes indicate that signals from that sensor location were used for the algorithm. These results are equivalent to Figure 2 but depicted here to visualize the performance of each specific sensor combination.

The algorithm workflow was first performed using all our candidate wearable sensor signals; we termed this the distributed sensor algorithm. Next, to evaluate the feasibility of using a reduced number of sensors for estimating lumbar moments, we developed additional algorithms using a reduced number of sensor signals (termed reduced sensor algorithms). While we explored 10 candidate wearable sensors (R/L pressure insoles, R/L foot IMUs, R/L shank IMUs, R/L thigh IMUs, pelvis IMU, trunk IMU), when iterating through potential reduced sensor algorithms, we assumed that a final solution would have symmetrical bilateral sensors (e.g., if the wearable included a right insole, then it would also include a left insole). Thus, our 10 candidate wearable sensors actually corresponded to 6 candidate sensor locations: trunk, pelvis, thigh, shank, and foot IMUs, and the pressure insoles. The algorithm workflow was repeated to develop 62 additional algorithms that each used a reduced set of 1 to 5 sensor locations (see Figure A1 of Appendix A for an overview of all the combinations).

#### 2.3.4. Algorithm Evaluation

We evaluated the accuracy of different sensor combinations in two stages. First, we computed the coefficient of determination (r^2^) to identify the most promising reduced sensor combinations and computed relative wearable sensor signal importance to identify the most important sensors. Then, we identified promising or interesting sensor combinations, reviewed wearable algorithm results using scatter plots and participant-specific results, and computed additional accuracy metrics to better understand the performance and limitations of each sensor combination.

We computed r^2^ for each participant across all time samples [20] for all candidate sensor combinations. Based on our prior work on wearables for musculoskeletal load monitoring [12,21], we have found r^2^ to be useful for this initial sensor combination selection process (i.e., down selection from 62 sensor combinations here) because it provides insight into how well wearable estimates correlate with lab-based gold-standard estimates across the full range of lumbar moments observed. This research is early stage, so there is no precise r^2^ threshold that we can define as the minimum viable, but to benchmark high algorithm accuracy we used r^2^ > 0.8 as a threshold for promising solutions.

As a complementary analysis to evaluate which sensors were most important for algorithm estimates, we applied the permutation feature importance method [22]. Feature importance values represent the drop in model accuracy (∆r^2^) when an input signal is randomly shuffled, with larger values indicating that the algorithm is more dependent on that signal. Of note, the permutation feature importance method was used rather than the impurity-based feature importance approach because the latter approach had some undesirable biases (e.g., favoring high cardinality features) and is not supported with histogram-based estimators.

Once a subset of promising sensor combinations was identified, we inspected participant-specific results with scatter plot data to understand the performance and limitations of each. We were particularly interested in how each sensor combination performed across the range of lumbar moment magnitudes observed (e.g., did certain sensor combinations perform better at low magnitudes vs. high magnitudes). We also computed the root mean square error (RMSE). In this data set, most samples are at relatively low lumbar moment magnitudes, but larger moments are the most damaging and dangerous to musculoskeletal tissues. We therefore also looked specifically at algorithm performance constrained to higher lumbar moments using mean absolute percent error (MAPE). We leveraged the benefits of both relative (r^2^ and MAPE) and absolute (RMSE) accuracy metrics, along with biomechanics knowledge of key factors that influence cumulative damage and overexertion injury risk, to make informed suggestions about using wearable sensors to monitor back loading across work-relevant lifting tasks.

## 3. Results

### 3.1. Results from Idealized Wearable Sensors

**Figure 2:**
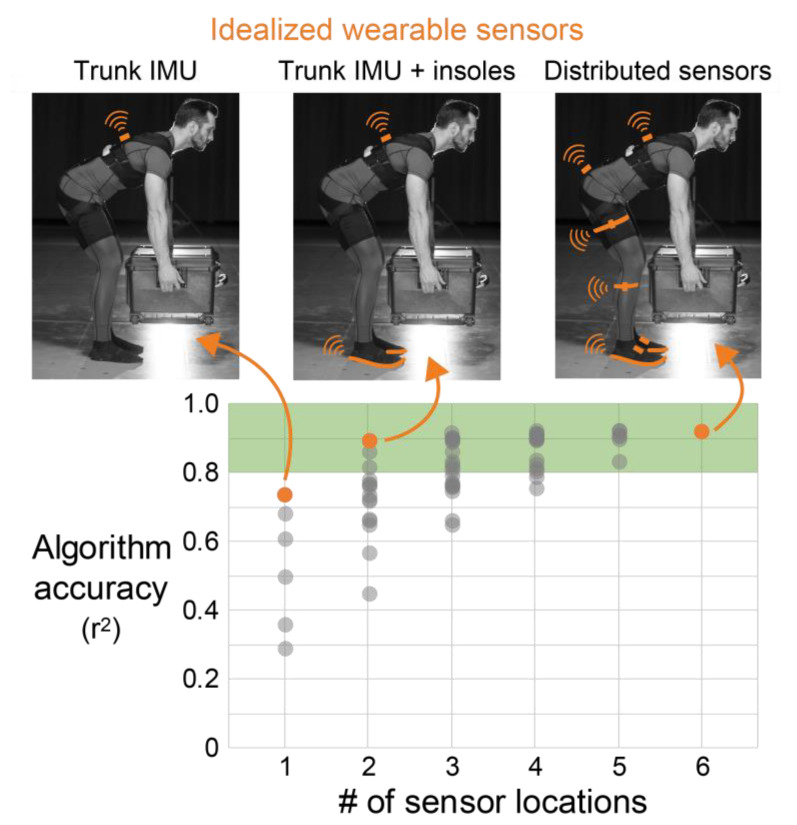
Maximum algorithm accuracy increased with number of sensor locations. Average accuracy using idealized wearable sensors summarized here using the average coefficient of determination (r^2^) across all participants. Orange dots represent the distributed sensor algorithm (**right**) and the highest accuracy algorithms using 1 (**left**) and 2 (**center**) sensor locations. All the algorithms here were developed with idealized wearable sensor signals and the target was lumbar extension moment. The top three algorithms using one and two sensor locations are reported in Table 3. A detailed summary of all the algorithm accuracies and the exact sensor combinations for each algorithm is included in Figure A1.

As expected, the maximum algorithm accuracy increased with the number of sensor locations (Figure 2). There were no single sensor solutions that yielded r^2^ > 0.8 (i.e., coefficient of determination greater than 0.8 between idealized wearable sensor algorithm estimates and lab-based lumbar moment estimates). However, there was a noticeable jump in accuracy when moving from one to two sensor locations (maximum r^2^ = 0.74 to r^2^ = 0.89, Figure 2, Table 3). When increasing the number of sensors beyond two locations, there were only small additional improvements in the maximum algorithm accuracy (from r^2^ = 0.89 using two sensor locations to r^2^ = 0.92 using all six sensor locations, the maximum number of distributed sensor locations in this study).

**Figure 3:**
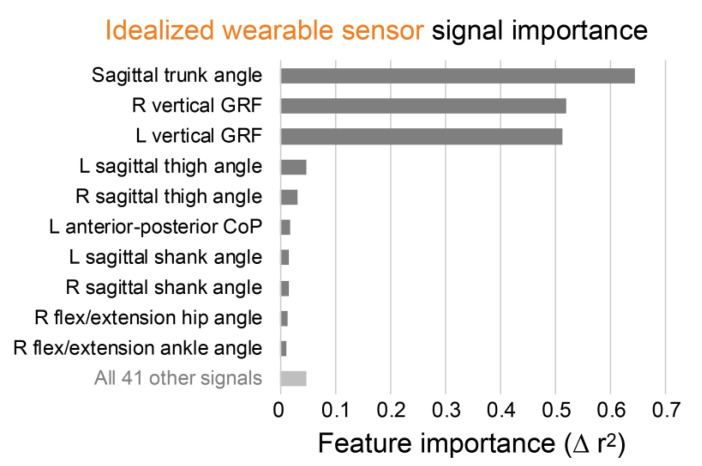
Sagittal trunk angle and vertical GRFs are the most important signals for estimating lumbar moments. Signal importances are from the idealized wearable sensor algorithm for estimating lumbar extension moments. R = right; L = left.

The two most important signals for estimating lumbar extension moments identified during algorithm development were sagittal trunk angle and vertical GRFs (Figure 3). Consistent with this, the best solution using two sensor locations is the one that combined a trunk IMU and pressure insoles (r^2^ = 0.89, Figure 2, Table 3). This combination was of highest interest to us because of its potential to be practical and accurate.

**Figure 4:**
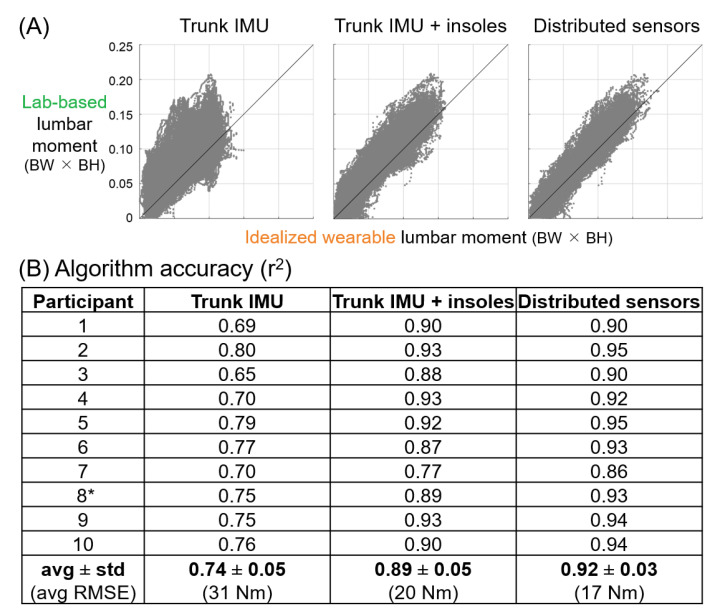
Algorithm accuracies for three idealized wearable sensor algorithms. (**A**) Lab-based (gold-standard) lumbar extension moment vs. idealized wearable algorithm estimates of lumbar moment for all time samples for an example participant (participant 8*). Positive moments correspond to lumbar extension moments. A line with a slope of one is added to visualize a perfect correspondence between lab-based and wearable estimates. BW × BH = body weight × body height. (**B**) Coefficient of determination (r^2^) for each participant. Average results (avg, bottom) are equivalent to accuracies in Figure 2. The trunk IMU algorithm was less accurate than the trunk IMU and pressure insoles algorithm, and than the distributed sensors algorithm (*p* < 0.001 and *p* < 0.001, respectively, based on Wilcoxon signed-rank test of the k-fold cross validation accuracy results). Comparing accuracies from the trunk IMU and pressure insoles algorithm vs. the distributed sensors algorithm yielded *p* = 0.054. Average RMSE was converted into units of Nm (using mean participant height and weight) and included for reference.

The trunk IMU (alone) and fully distributed sensor set were also of interest for further analysis. The trunk IMU provides a point of reference for the potential accuracy of existing commercial wearables that use a single IMU to monitor lumbar loading, while the distributed sensor set provides insight on accuracy gains with higher instrumentation coverage. Therefore, we report participant-specific results and additional accuracy summary metrics (RMSE and MAPE) for these different sensor combinations (Figure 4). 

The distributed sensor algorithm resulted in an average RMSE of approximately 17 Nm (Figure 4B), equivalent to about a 241 N (0.3 BW) error in spine compression force (assuming a 7 cm lumbar extensor muscle moment arm, [23]). The trunk IMU and pressure insole algorithm resulted in an average RMSE of approximately 20 Nm, equivalent to about a 282 N (0.4 BW) error in spine compression force. The trunk IMU algorithm resulted in an average RMSE error of approximately 31 Nm, equivalent to about a 444 N (0.6 BW) error in spine compression force. As one additional point of reference, the NIOSH Lifting Equation recommends limiting spine compression force to less than 3400 N (4.4 BW), so these RMSE values are about 7%, 9%, and 14% of this limit, respectively. Given the sensitivity of MAPE when target values are close to zero, we also computed the MAPE for all samples when the target load metric was greater than 0.05 BW × BH (which encompassed about half of all time samples of data for each participant). The average MAPE for the upper range of lumbar moments was 13%, 15%, and 25% for the distributed sensor, trunk IMU and pressure insole, and trunk IMU algorithms, respectively.

We also observed that if the trunk IMU were substituted with thigh IMUs, then correlations only decreased slightly from r^2^ = 0.74 to r^2^ = 0.68 with a single sensor, and from r^2^ = 0.89 to r^2^ = 0.86 for the two sensor combination (Table 3). If the trunk IMU were substituted with a pelvis IMU, then the correlations decreased slightly more from r^2^ = 0.74 to r^2^ = 0.61 with a single sensor, and from r^2^ = 0.89 to r^2^ = 0.81 for the two sensor combination (Table 3). All of the two sensor location solutions that achieved r^2^ > 0.8 included GRFs from pressure insoles.

Participant-specific results (Figure 4) corroborated and strengthened the average results (Figure 2 and Figure 3, Table 3). For instance, all ten participants exhibited high algorithm accuracies (r^2^ ranging from 0.86 to 0.95) using the distributed (six sensor location) algorithm. When moving from a single trunk IMU to using a trunk IMU and pressure insoles, every participant exhibited an increase in r^2^ value (Figure 4). Scatter plot data for each participant indicated that the improvement in r^2^ going from one to two sensor locations was driven by both a decrease in the variation of data about the unity regression line and improved estimates at higher magnitude lumbar moments (see the example participant data in Figure 4A). When moving from two to six sensor locations, the variation in data about the regression line decreased more, but only slightly (Figure 4A). We also note that for two participants (numbers 1 and 4), going from two to six sensor locations did not increase r^2^ at all (Figure 4B). 

### 3.2. Results from Real Wearable Sensors

**Figure 5:**
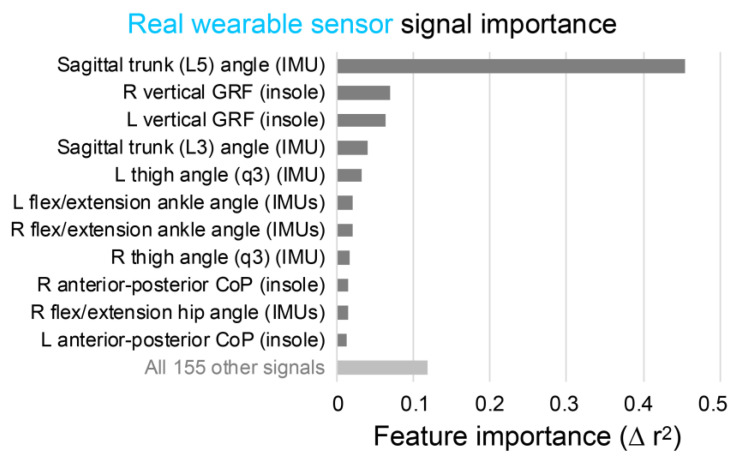
Sagittal trunk angle and vertical GRFs are the most important signals for estimating lumbar moments, consistent with the findings from idealized wearable sensor analysis in Figure 3. Signal importances here are from the real wearable sensor algorithm for estimating lumbar extension moments. R = right; L = left.

**Figure 6:**
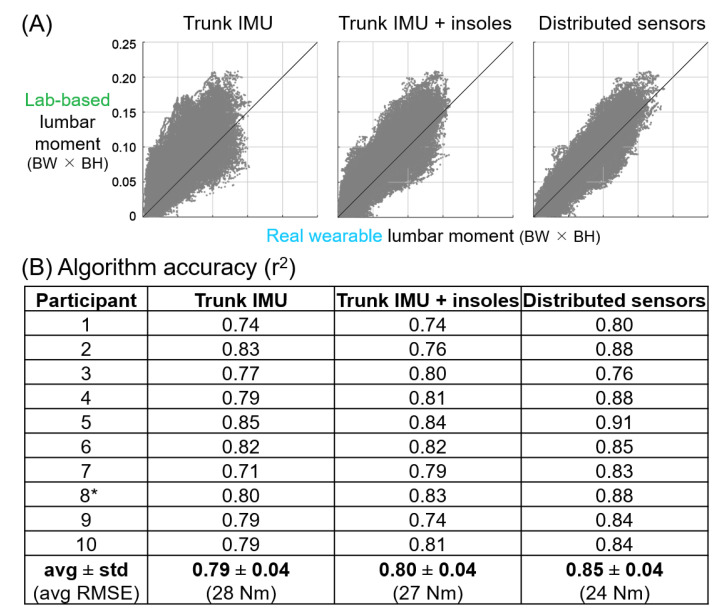
Algorithm accuracies for three different real wearable sensor combinations. (**A**) Lab-based (gold-standard) lumbar moment vs. real wearable sensor algorithm estimates of lumbar moment for all time samples for an example participant (participant 8*). Positive moments correspond to lumbar extension moments. A line with a slope of one is added to visualize a perfect correspondence between lab-based and wearable estimates. BW × BH = body weight × body height. (**B**) Coefficient of determination (r^2^) for each participant. Both reduced sensor algorithms yielded accuracies that were lower than the distributed sensor combination (*p* = 0.011 and *p* = 0.014 for the trunk IMU, and trunk IMU and insole algorithms, respectively; based on Wilcoxon signed-rank test of the k-fold cross validation accuracy results), but the accuracy of the two reduced sensor algorithms shown here were not different from each other (*p* = 0.571).

Figure 5 is analogous to Figure 3, and Figure 6 is analogous to Figure 4, except that Figure 5 and Figure 6 are based on algorithms using real wearable sensors rather than idealized wearable sensors. Real wearable sensor results confirm that the most important sensor signals for estimating lumbar extension moments are sagittal trunk angle from a trunk IMU and vertical GRFs from pressure insoles (Figure 5). However, it is noteworthy that the trunk angle signal importance was much higher than the vertical GRFs in the analysis using real wearable sensors (Figure 5), whereas with idealized signals these signal importances were of similar magnitude (Figure 3).

The participant-specific results (Figure 6) again corroborated and strengthened the average results from real wearable sensor algorithms. Compared to idealized wearable sensor algorithms, there was no discernible increase in r^2^ value when moving from one sensor location (trunk IMU, r^2^ = 0.79) to two sensors locations (trunk IMU and pressure insoles, r^2^ = 0.80). The increase in r^2^ from two to six sensor locations also remained relatively small, similar to what was observed in the idealized wearable sensor analysis (Figure 4).

### 3.3. Comparison of Results from Idealized versus Real Wearable Sensors

**Figure 7:**
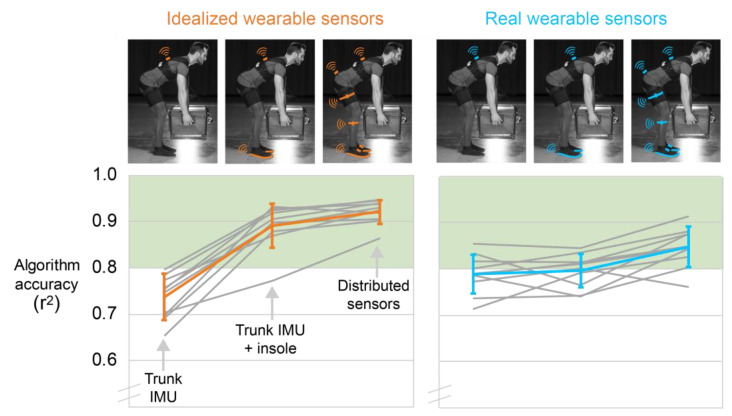
Side-by-side comparison of algorithm performance using idealized versus real wearable sensor signals. Gray lines are each of the 10 participants’ accuracy results, and colored lines are the average (and standard deviation) across the 10 participants. Results using idealized wearable sensors are shown on the left (orange) and results using real wearable sensors are shown on the right (blue).

Figure 7 provides a side-by-side comparison of algorithm performance using idealized versus real wearable sensor signals. These plots are visualizations of the tabular results reported in Figure 4B and Figure 6B and provided for clarity and to assist with interpretation. The key takeaway is that while the idealized wearable sensor analysis resulted in a noticeable jump in accuracy when moving from one to two sensors, a similar improvement was not observed in the real wearable sensor analysis (Figure 7). The Discussion section digs into why.

## 4. Discussion

These findings indicate that there is strong potential to use a small number of wearable sensors to create a portable tool for the practical and accurate monitoring of low back loading over a broad range of manual material handling tasks. We characterized the performance of over 60 different wearable sensor combinations and algorithms. The solution we found to be most promising combines signals from sensors at two body locations (an IMU on the trunk and pressure insoles under the feet) with a Gradient Boosted Decision Tree algorithm. While idealized wearable sensor results demonstrated promising proof-of-concept, the analysis of real wearable sensor signals revealed that to achieve accurate lumbar moment estimates in the real world, the key technological challenge will be to optimize force estimates and minimize variability from the pressure insoles. With further development and validation, we believe that this type of wearable solution has the potential to transform how ergonomic assessments are performed in industry, to enhance the quality, quantity, and efficiency of occupational data collection, and to expand opportunities for personalized, continuous monitoring of low back injury risk. For example, time-series lumbar moments could be partitioned into individual lift/bend cycles and the magnitude and frequency of loading on the low back could be automatically input into ergonomic assessment tools like LiFFT to estimate overexertion injury risk. Below we discuss the major technical findings from this exploratory research, along with alternative solutions, key challenges, and new opportunities for advancement.

### 4.1. Which Wearable Sensors and Locations Are Most Important?

The trunk IMU and pressure insoles were identified in all analyses as together being the most important sensors for monitoring lumbar extension moments (Figure 2, Figure 3 and Figure 5; Table 3). These results match our biomechanics intuition given that lumbar moment is strongly influenced by the weight of the object being lifted (which can be captured by pressure insoles) and by upper-body posture (which can be estimated with an IMU on the trunk).

Interestingly, the trunk IMU could be replaced with thigh IMUs or a pelvis IMU with relatively little degradation in accuracy (Table 3). Of note, the reason that the thigh and pelvis IMU signals appear to have a low importance in Figure 3 and Figure 5, but can actually be useful substitutes for the trunk IMU is because they are highly correlated with other signals and because of how the feature importance method works (see Methods). It is valuable to acknowledge these other alternatives because some sensor locations may be preferred for certain applications; for instance, a fall protection harness manufacturer may be able to integrate an IMU more easily on the trunk near the D-ring or on the thighs using the leg loops, whereas for a tool belt manufacturer it may be preferable to integrate the IMU at the waist. In contrast, there was no substitute for the pressure insoles, which provide unique force data that helps to distinguish if the person is lifting a heavy object vs. a light object vs. no object at all and just bending forward. In theory, object mass could be obtained using sensors beyond those we tested (e.g., measured directly using force-instrumented gloves, or estimated indirectly via muscle EMG), but these again introduce added complexity and practical implementation challenges that may be barriers to adoption for many applications.

We observed that using two sensor locations (trunk IMU and pressure insoles) sacrificed minimal accuracy compared to using more sensor locations (e.g., all six distributed sensor locations, Figure 4 and Figure 6). This supports the idea that it may be possible to use a relatively small subset of sensors to make workplace implementation more practical, while still obtaining accurate estimates of back loading. These findings also demonstrate that more sensors, or more widely distributed sensors, should not be assumed to result in substantially more accurate musculoskeletal load monitoring tools. For monitoring lumbar loading during manual material handling, there appears to be a sweet spot for accuracy and practicality that involves using pressure insoles and a single IMU.

### 4.2. What Types of Algorithms Work Well for This Sensor Data Fusion?

All the results presented here were developed using Gradient Boosted Decision Tree algorithms. We found this type of algorithm to work well during early exploration of the data. Within the Gradient Boosted Decision Tree framework, we utilized the histogram-based decision tree building algorithm, as it significantly reduces the training time with larger datasets (>10k samples), but did not noticeably degrade prediction performance of our algorithms, compared to traditional Gradient Boosted Decision Trees. Using this approach, input signal values are separated into bins, reducing the computational complexity of splitting decisions and efficiently leveraging parallel computational resources [24].

In a pilot data analysis, we also explored other categories of algorithms/models, including generalized linear models, ensemble methods (random forests), shallow neural networks (2 hidden layers), and support vector regression. While most of these methods (linear, support vector regressions, forests) resulted in comparable prediction results to each other, Gradient Boosting consistently provided more accurate estimates in our preliminary data sets. Additionally, some of these methods (most notably, support vector regression) did not scale well with a large number of data points and became prohibitive to train.

We did not have success with traditional neural network models. This may have been because of insufficient number of layers, nodes, or the chosen activation functions. We note that the hyperparameter space for neural networks is significantly larger than for the other methods we tried. We provide this brief commentary on the explored set of machine learning algorithms for this problem domain to share our initial experiences. Our review and evaluation of alternative algorithm approaches is not exhaustive and there are certainly other applicable AI-based or statistical methods beyond this initial study. Such promising candidates include convolutional neural layers and recurrent neural networks, which may be interesting to explore in the future. 

### 4.3. How Accurately Can We Monitor Low Back Loading during Manual Material Handling Tasks?

The idealized results demonstrate the potential for a small number of sensors to provide accurate estimates of low back loading. Using a trunk IMU and pressure insoles resulted in lumbar moment estimates that were strongly correlated with lab-based lumbar moments (r^2^ = 0.89, Figure 4B). This solution performed well across the broad range of tasks and lumbar moment magnitudes captured (Figure 4A). The RMSE and MAPE accuracy results corroborated that this wearable sensor approach is very promising. The RMSE corresponds to less than 10% of the peak lumbar moments during heavy lifting. For context, we found that using just two sensor locations (trunk IMU and pressure insoles) during about 400 different material handling tasks exhibited similar levels of accuracy (r^2^ = 0.89 and RMSE = 20 Nm) as those reported in [8], which combined 8–17 IMUs and force-sensing shoes to estimate lumbar moments during 4 tasks that involved lifting and carrying a 10 kg box (r^2^ = 0.93 and RMSE < 20 Nm).

The real wearable results highlighted the technological key to realizing accurate estimates of back loading in the real world. Combining a real wearable trunk IMU and pressure insoles resulted in lower average accuracy than with the idealized wearable sensors (e.g., r^2^ = 0.80 vs. r^2^ = 0.89), and only marginal benefits over a real trunk IMU alone (r^2^ = 0.80 vs. r^2^ = 0.79). This appears to be due to variability in insole force estimates compared to vertical forces from idealized wearable sensors (i.e., from lab-based force plates, Figure A5). In contrast, we found that trunk orientation from the real wearable sensor (trunk IMU) was a very strong indicator of trunk orientation from idealized wearable sensors (lab-based optical motion capture), with low variability (Figure A5). Together, this seems to explain why GRFs were of similar importance as the trunk IMU when using the idealized wearable sensors (Figure 3) but of much lower importance when using the real wearable sensors (Figure 5). A key technological priority should be to reduce the variability in the insole force estimates. The good news is that there are various ways to improve these force estimates through advances in signal processing, calibrations, and sensor hardware, or via the optimization of sensors for pressure/force magnitudes expected in certain tasks such as material handling. As the variability in insole forces is reduced, the accuracy of algorithms developed using real wearable sensors will approach the accuracy observed using the idealized wearable sensors. We confirmed this to be true by replacing the real pressure insole data with idealized pressure insole data during algorithm development and finding lumbar moment estimates to have similar accuracy to our idealized wearable sensor algorithms.

These insights highlight the benefits of using idealized signals when exploring new wearable sensor solutions. If we used real wearable sensors alone, we may have concluded that pressure insoles do not improve back loading estimates compared to a single wearable trunk IMU. In actuality the pressure insoles provide unique and highly valuable force data (Figure 2 and Figure 3, Table 3) that can help distinguish when someone is lifting a heavy object vs. simply bending forward, and that can greatly improve capabilities for monitoring trends in low back loading (particularly at higher magnitudes). Overall, our complementary analyses, evaluating accuracies across a range of reduced sensor algorithms for both idealized and real wearable sensors, and ranking signal importances, provides a systematic and effective approach to identifying key sensor signals and promising wearable sensor combinations.

### 4.4. Benefits and Drawbacks of Single Wearable Sensor Solutions

The results demonstrate that a single IMU solution can perform reasonably well for estimating lumbar moments (Table 3). The practical benefits were described in the Introduction (e.g., relative simplicity for workplace implementation). The trunk IMU, and to a slightly lesser extent the thigh and pelvis IMUs, provided moderately high correlation coefficients up to r^2^ = 0.74 in idealized wearable sensor analysis, and up to r^2^ = 0.79 in the real wearable sensor analysis. The reason for the slightly stronger correlations with real wearable sensors for the trunk IMU algorithm is unknown, but may be due to a richer set of candidate signals that we input into the real vs. idealized wearable algorithms (see Table 1 vs. Table 2), which included additional spine segment and joint angle estimates from the Xsens functional skeleton calibration, and IMU accelerations and velocities. These results suggest that commercial wearables that place an IMU on one of these segments (trunk, pelvis, or thighs) are at least monitoring the types of signals that can be correlated with lumbar moments (with proper algorithm development and training).

**Figure 8:**
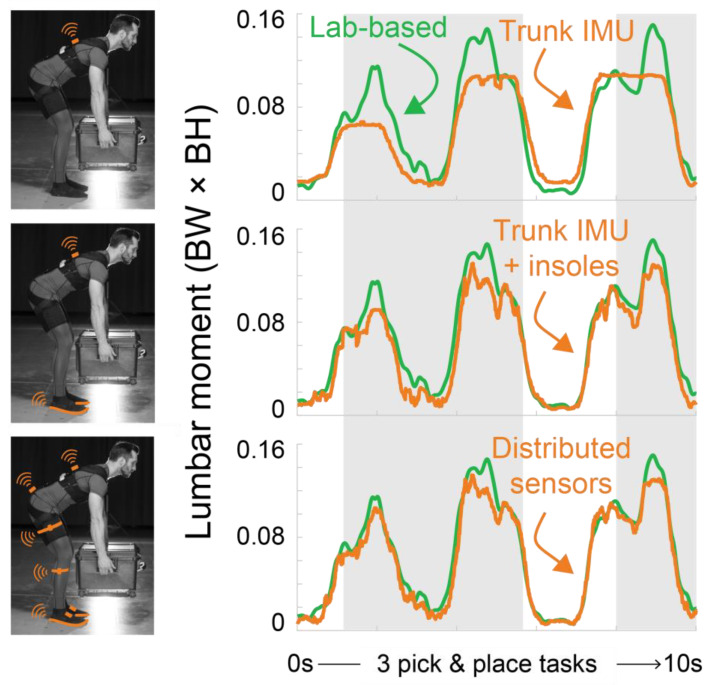
Single IMU wearable does not capture key trends and peaks in lumbar loading when objects are lifted. Time-series lab-based lumbar extension moment (green) and idealized wearable algorithm moments developed with three different idealized wearable sensor combinations (orange). Shown is an example lifting task from the hundreds of manual material handling tasks performed for an example participant; 3 pick and place task cycles with a 10 kg box shown. While we only show a subset of time-series results here, we observed similar algorithm accuracy trends across the broad range of manual material handling tasks collected. Gray areas are approximately when the participant was holding the 10 kg box, white areas are when the participant had no object in their hands. The trunk IMU tends to perform worse when the box is being held or lifted, whereas the trunk IMU plus pressure insoles, and distributed sensors, are able to better track key lumbar loading trends (gray areas). BW × BH = body weight × body height.

The critical drawback of single IMU wearables is that they fail to capture increases in lumbar loading when different objects are lifted, and as a result they tend to perform worse for higher lumbar moments, which unfortunately are the instances of highest ergonomic interest since these are most damaging to musculoskeletal tissues. This accuracy limitation at higher magnitudes is evident in plots of time series lumbar moments. For example, the trunk IMU algorithm does not capture the increase in low back loading peaks when a participant is picking and placing a 10 kg box (gray areas in Figure 8). In contrast, these elevated back loads from the handheld mass are captured by solutions that use sensors at multiple locations that include pressure insoles along with at least one IMU (Figure 8). The time-series plots show a representative lifting task, while the scatter plots and tables presented in the Results provide comprehensive results from all the participants and across all the manual material handling tasks collected.

**Figure 9:**
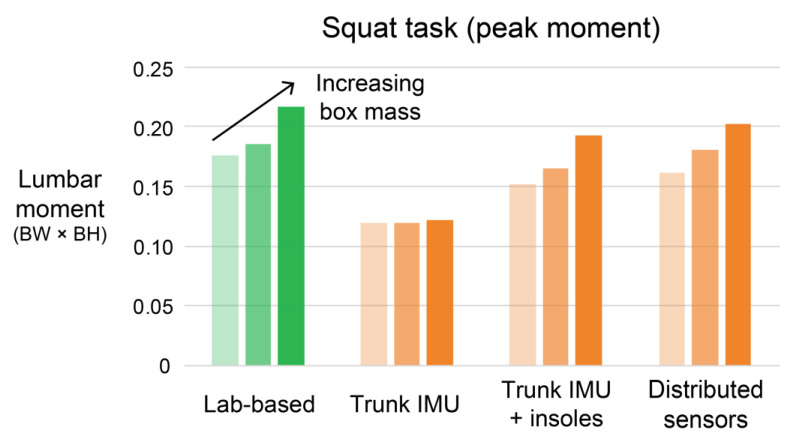
Single IMU wearable does not capture increases in lumbar loading when heavier objects are lifted. Shown is an illustrative example from one participant: peak lumbar moment of squat tasks when increasing box masses are lifted (10 kg, 15 kg, 23 kg are shown). The trunk IMU and insole algorithm, and also the distributed sensor algorithm, captures the trend of increasing lumbar moment with heavier object mass. However, the trunk IMU algorithm does not; it predicts a similar peak moment with each lift regardless of the mass being lifted. BW × BH = body weight × body height.

As another example, lifting objects of increasing mass with similar body posture causes an increase in peak lumbar moments (Figure 9). Using a trunk IMU alone completely misses the trend of increasing low back loading when individuals adopt similar trunk orientations (i.e., postures) for each lift, while using a solution that includes both pressure insoles and an IMU captures these increasing back load trends (Figure 9). These results confirm our expectations from the Introduction: while a single IMU (on the trunk, or elsewhere) may provide a reasonable estimate of back loading (or trends in loading) due to changes in general body posture, the estimation accuracy is compromised when objects of differing mass are handled or when other external forces are applied to the body (e.g., during pushing, pulling, or leaning). It may also be possible to use the trunk IMU plus pressure insoles combination during initial assessment of each worker, or intermittently over time, in order to better calibrate the trunk IMU (alone) for each worker—in effect supplementing the minimal single senor solution to improve accuracy and personalization. Of note, using the pressure insoles alone yielded fairly poor accuracy (Figure A1), again highlighting the benefits of fusing data from multiple sensor locations.

In short, caution should be taken when using a single wearable (on any body segment) to monitor low back loading, particularly in situations where external forces are variable, or when object masses being handled are not manually input (or otherwise accounted for) in algorithms. Further exploration is warranted to understand the implications of single IMU sensor accuracy within the context of risk assessment tool sensitivity, and to understand the validity of using single wearable sensor solutions for different types or subsets of manual material handling tasks. Wearable solutions that fuse data from multiple sensor locations (e.g., trunk IMU and pressure insoles) are expected to provide more accurate and reliable ways to automate ergonomic assessments or provide continuous daily risk monitoring for material handling jobs that involve lifting objects of varying weight; albeit with slightly more implementation complexity due to more sensor modalities, and presuming the variability in pressure insole force estimates can be adequately reduced.

### 4.5. Lateral Bending Lumbar Moment Can Also Be Estimated with a Trunk IMU and Pressure Insoles

**Figure A2:**
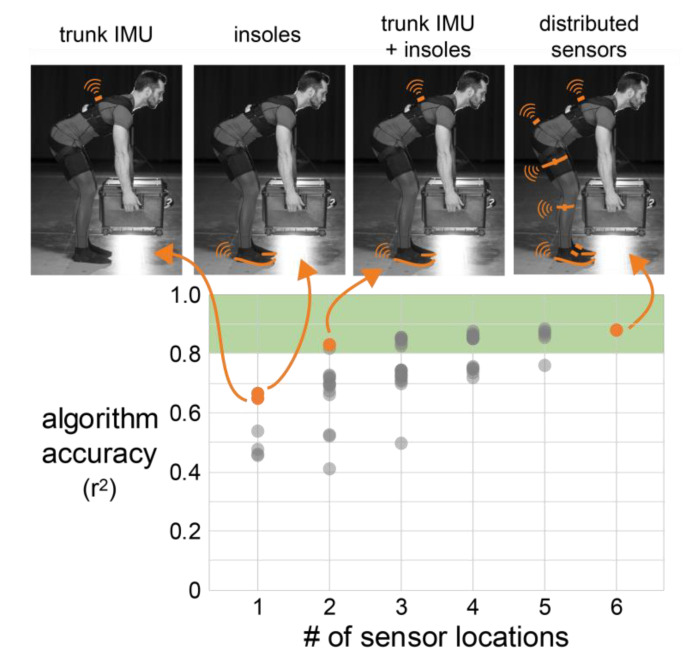
Combining a trunk IMU and pressure insoles is also promising for estimating lumbar lateral bending moment. Average accuracy using idealized wearable sensors summarized here using the average coefficient of determination (r^2^). As with estimating lumbar extension moments (Figure 2), the maximum algorithm accuracy increased with number of sensor locations. Orange dots represent the distributed sensor algorithm (6 sensor locations), and a subset of algorithms using 1 and 2 sensor locations. All the algorithms here were developed with idealized wearable sensor signals and the target was the lumbar lateral bending moment.

**Figure A3:**
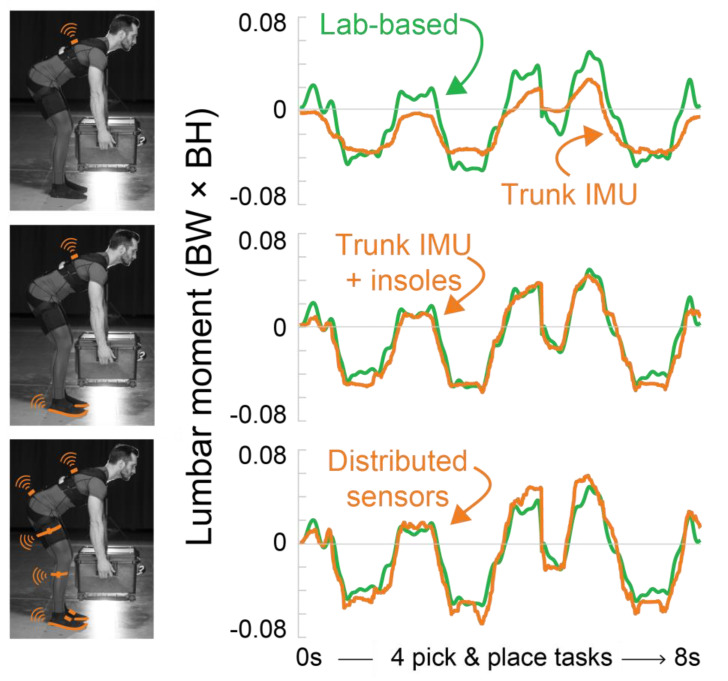
Representative example of lumbar lateral bending moment estimates based on different wearable sensor combinations. Shown are the lab-based (green) and algorithm-estimated (orange) lateral bending moments for three different sensor combinations. Depicted is a subset of the hundreds of manual material handling tasks performed for an example participant; 4 pick and place task cycles with a 5 kg box shown. These results were similar to those observed when estimating the lumbar extension moment (Figure 8): the single IMU wearable did not well estimate the higher magnitude lateral bending moments, but combining a pressure insole with at least one IMU improved these estimates. All the algorithms here were developed with idealized wearable sensor signals and the target was lumbar lateral bending moment. BW × BH = body weight × body height.

**Figure A4:**
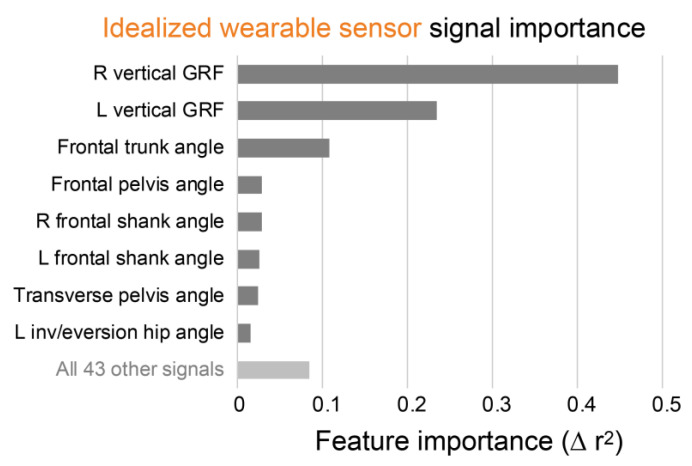
Vertical GRFs and frontal trunk angle are the most important signals for estimating lumbar lateral bending moments. Signal importances are from the idealized wearable sensor algorithm for estimating the lumbar lateral bending moment. Note that these signals can be obtained from the same two sensors (trunk IMU and pressure insoles) that we identified as being the most important for estimating the lumbar extension moment. R = right; L = left.

**Figure A5:**
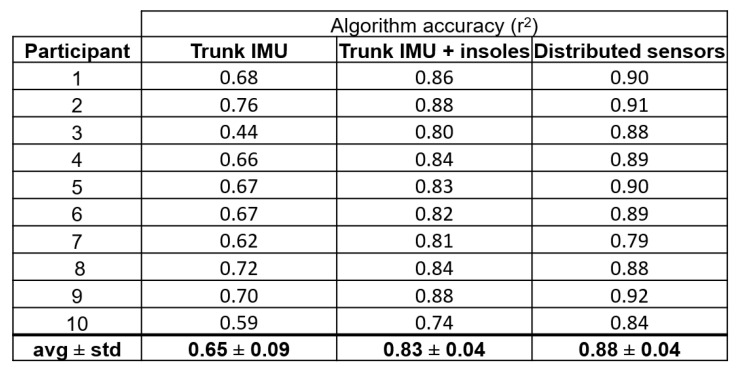
Algorithm accuracies when estimating the lumbar lateral bending moment. Shown are the participant-specific and average (avg) accuracy results from three different subsets of idealized wearable sensors: trunk IMU, trunk IMU and pressure insoles, and all distributed sensors. Accuracy is reported as the coefficient of determination (r^2^) across all time samples for a given participant.

Lumbar extension moments have been shown to be a key metric for monitoring cumulative damage to the low back and resulting injury risk (see Introduction). However, there are also opportunities to provide a broader, multifactorial assessment of injury risk by monitoring other musculoskeletal loading metrics with wearables. One additional metric of interest to us was lumbar lateral bending moment, as increases in lateral bending moment contribute to increases in back muscle and disc compression forces [25], which influence cumulative damage to the low back. We therefore repeated the same algorithm development and evaluation process using the idealized wearable sensor data from this study, but using time series lumbar lateral bending moment as the target metric (Figure A2, Figure A3, Figure A4 and Figure A5). Encouragingly, signals from the same set of wearable sensors (trunk IMU and pressure insoles) that were identified as most important for estimating lumbar extension moments were also the most important for estimating lateral bending moments (Figure A4). Similar to our analysis of the lumbar extension moment, a single trunk IMU algorithm did not capture all trends in lateral bending moment, namely when the user held and moved objects of differing mass lateral to their body (Figure A3). The trunk IMU algorithm resulted in an average accuracy of r^2^ = 0.65 (Figure A5). Combining the pressure insoles with the trunk IMU increased the accuracy to r^2^ = 0.83 (Figure A5). This again demonstrates how a small set of wearable sensors (trunk IMU and pressure insoles) could provide a practical and accurate tool for monitoring low back loading (due to both lateral and extension moments), with only a relatively small reduction in accuracy compared to the full set of distributed sensors tested (r^2^ = 0.88, Figure A5).

### 4.6. Limitations and Future Opportunities

Given the exploratory nature of developing next generation wearables, there were many interesting additional areas of research that were beyond the scope we chose to evaluate in this study. Numerous other candidate wearable sensors and emerging technologies, signal processing techniques, machine learning algorithms, and musculoskeletal metrics of interest could be explored in future studies. Additionally, while we focus on evaluating a tool for monitoring low back loading in a workplace environment, there are many other exciting research and clinical applications of a low back monitoring tool. For example, a similar wearable solution might be used in a clinical or home setting to monitor patients during post-injury or post-surgery rehabilitation, track their progress, or assist with return-to-work decisions.

Within the scope of this study, we note some limitations of our approach. First, the real wearable sensors used were research-grade instrumentation. Implementing algorithms on consumer-grade hardware, or any other hardware platform not tested here, would require additional algorithm calibration, validation and evaluation. Second, the number of participants tested was informed by our prior studies combining wearable sensors and machine learning [12,21], but this kind of exploratory (non-hypothesis-driven) research is not amenable to traditional sample size calculations. The consistency of results for individual participants using the k-fold validation analysis suggests that our sample was adequate, but we acknowledge that our understanding of how much data is enough to identify promising wearable monitoring tools using diverse machine learning techniques is continuing to evolve. Third, we did not use sensors to monitor the location of the object being lifted relative to the body (e.g., spine). Although this distance could be estimated by tracking multiple segments of the arms, we choose not to do this for reasons of simplicity and practicality. For now, adding this complexity seems unnecessary given that the simpler trunk IMU plus pressure insole solution presented here already shows strong potential for estimating lumbar moments. Fourth, we focused on load monitoring as a key risk factor for low back disorders, but it is worth reminding that sensors like the trunk IMU capture other data such as twisting (spine rotation) and trunk acceleration/deceleration, which can also be useful and complementary for injury risk assessment. Fifth, we focused on how lumbar moments could be used as direct inputs into the LiFFT risk assessment tool. However, we should note that lumbar moments (and other wearable metrics like trunk orientation) could alternatively be input into other tools or models such as the NIOSH Lifting Equation. However, the NIOSH Lifting Equation requires some additional inputs such as a “Coupling” factor. Fully automating wearable risk assessment using the NIOSH Lifting Equation seems feasible, but would require some additional assumptions, modeling, and validation. Sixth, algorithms were developed and evaluated on a broad range of movement tasks we identified as representative of many manual material handling jobs performed in workplace environments. The efficacy of using a trunk IMU and pressure insoles to monitor low back loading for other tasks or jobs outside of those tested would require additional validation and evaluation. To our knowledge, this is one of the largest databases ever collected from synchronized laboratory instrumentation and wearable sensors in this ergonomics and material handling domain. As such, we plan to use this dataset for future secondary analysis, and to make it available to other researchers interested in exploring additional research questions.

## 5. Conclusions

Here, we present a promising wearable solution for the practical, automated, and accurate monitoring of low back loading during manual material handling. We found that two key sensors for accurately monitoring low back loading are a trunk IMU and pressure insoles. Using signals from these two sensors together with a Gradient Boosted Decision Tree algorithm has the potential to provide a practical (relatively few sensors), accurate (up to r^2^ = 0.89), and automated way (using wearables) to monitor time series lumbar moments across a broad range of material handling tasks. The trunk IMU could be replaced by thigh IMUs or a pelvis IMU without sacrificing much accuracy, but there was no practical substitute for the pressure insoles. The key to realizing accurate lumbar load estimates with this approach in the real world will be optimizing force estimates from pressure insoles. This promising wearable solution has the potential to transform low back injury risk assessment, monitoring, and prevention in various industries.

## Figures and Tables

**Table 1:** Idealized wearable sensor signals. R = right; L = left.

Idealized Wearable Sensors	Idealized Wearable Sensor Signals	# of Signals
8 idealized IMUs (trunk, pelvis, R/L thigh, R/L shank. R/L foot) Segments (8): pelvis, trunk, R/L thigh, R/L shank. R/L foot Joints (7): lumbar, R/L hip, R/L knee, R/L ankle	XYZ segment kinematics (Euler angles)	24
	XYZ joint kinematics	21
Idealized pressure insoles (R/L)	3D force plate GRF transformed into foot’s coordinate frame and projected onto 1D normal force	2
	Force plate center of pressure transformed into foot’s X/Y coordinate frame	4
Total		51

**Table 2:** Real wearable sensor signals. R = right; L = left.

Idealized Wearable Sensors	Idealized Wearable Sensor Signals	# of Signals
8 IMUs (sternum, pelvis, R/L thigh, R/L shank. R/L foot) Segments (11): pelvis, L5, L3, T12, T8, R/L thigh, R/L shank. R/L foot Joints (10): L5S1, L4L3, L1T12, T9T8, R/L hip, R/L knee, R/L ankle	XYZ segment kinematics (Euler angles)	33
	Segment kinematics (quaternions)	44
	XYZ segment velocities	33
	XYZ segment accelerations	33
	XYZ joint kinematics	30
Pressure insoles (R/L)	Total normal force	2
	X/Y center of pressure	4
Total		179

**Table 3:** Algorithm accuracy for a subset of idealized wearable algorithms. Average accuracy for the distributed sensor algorithm and the top three algorithms requiring one or two sensor locations. Accuracies reported here correspond to data points in Figure 2 and Figure A1.

# of Sensor Locations	Idealized Wearable Sensor Combination	Algorithm Accuracy (r^2^)
6	Distributed sensors	0.92
2	Trunk IMU + insoles	0.89
2	Thigh IMUs + insoles	0.86
2	Pelvis IMU + insoles	0.81
1	Trunk IMU	0.74
1	Thigh IMUs	0.68
1	Pelvis IMU	0.61

**Figure A6:**
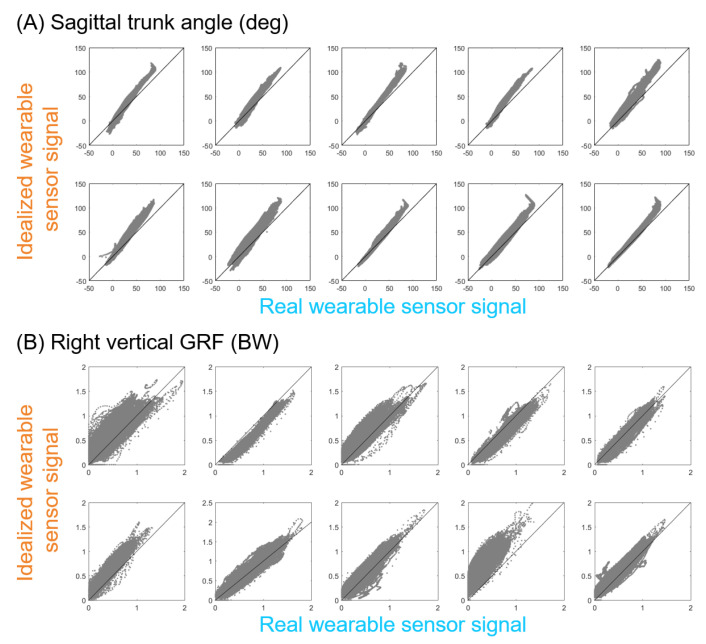
Trunk IMUs provided a relatively precise estimate of trunk orientation while pressure insoles provided a more variable estimate of vertical force. Ten scatter plots represent each participant and gray dots represent each time sample. Real wearable trunk orientation from an IMU correlated well with the idealized wearable trunk angle from lab-based motion capture (**A**), whereas the real wearable vertical force from pressure insoles did not correlate as well with the idealized wearable vertical force from lab-based force plates and exhibited a higher variability (**B**). A line with a slope of one is added to visualize a perfect correspondence between idealized and real wearable sensor signals. See Table 1 and Table 2 for details on idealized and real wearable sensor signals. deg = degrees; BW = body weight.

## Supplementary Materials

The following are available online at https://www.mdpi.com/1424-8220/21/2/340/s1: Video S1: Examples of manual material handling tasks performed in these experiments.
